# Burden of HIV and Syphilis: A Comparative Evaluation between Male Sex Workers and Non-Sex-Worker Men Who Have Sex with Men in Urban China

**DOI:** 10.1371/journal.pone.0126604

**Published:** 2015-05-11

**Authors:** Weiming Tang, Tanmay Mahapatra, Fengying Liu, Gengfeng Fu, Bin Yang, Joseph D. Tucker, Jinkou Zhao, Roger Detels

**Affiliations:** 1 Guangdong Provincial Center for Skin Diseases and STIs Control, No. 2 Lujing Road, Guangzhou, 510085, China; 2 University of North Carolina Project-China, No. 2 Lujing Road, Guangzhou, 510085, China; 3 Department of Epidemiology, School of Public Health, University of California Los Angeles, Los Angeles, CA, United States of America; 4 Jiangsu Provincial Central for Disease Prevention and Control, Nanjing, Jiangsu, China; 5 Monitoring and Evaluation Unit, The Global Fund to Fight AIDS, Tuberculosis and Malaria, Geneva, Switzerland; Fudan University, CHINA

## Abstract

**Background:**

The increasing burden of sexually transmitted infections (STIs) including HIV and syphilis among male sex workers (MSWs) is a major global concern. The aim of our study was to evaluate the difference between MSWs and non-commercial MSMs in China.

**Methods:**

During 2008-09, in a cross-sectional study, 2618 adult MSM were recruited through respondent-driven and snowball sampling from seven cities of China. Information regarding socio-demographics, risk behaviors, HIV-related knowledge and STI-related symptoms were collected and participants were tested for HIV and syphilis.

**Results:**

Among 2618 participating MSM, 9.97% sold sex to males. HIV prevalence was 7.45% (6.13% among MSWs and 7.59% among non-MSW MSM) and syphilis prevalence was 14.32% (10.73% for MSWs and 14.72% for non-MSW MSM). Compared to non-MSW MSM, MSWs were more likely to be younger (adjusted odds ratio: aOR = 0.91, 95% confidence interval: 95%CI=0.88-0.93), never married (aOR = 4.38, 95% CI = 2.38-6.80), less educated, heterosexual (aOR = 13.04, 95% CI = 6.08-27.95), less knowledgeable regarding HIV (aOR = 0.70, 95% CI=0.51-0.96), experiencing symptoms of STI (aOR = 2.16, 95% CI = 1.47-3.19), engaging in condomless vaginal intercourse (aOR = 2.16, 95% CI = 1.47-3.19) and less likely to engage in condomless anal intercourse (aOR = 0.62, 95% CI = 0.46-0.85).

**Conclusions:**

High HIV and syphilis prevalence warranted urgent intervention targeting MSWs as a separate sentinel group for efficient surveillance owing to their different distribution from non-MSW MSM. Although male sex workers and non-commercial homosexuals have similar rates of HIV and syphilis, MSWs have different characteristics which should be considered in designing intervention programs targeting them.

## Introduction

The high burden of HIV and syphilis among men who have sex with men (MSM) are major public health concerns worldwide and China is no exception [1–3]. Globally, Sub-Saharan Africa and North America have the highest HIV burden among MSM [3]. In China, HIV prevalence among MSM is approximately 6.0% [1]. As a subgroup of MSM, men who sell sex to males for money or goods (male sex workers, or MSWs, who may also purchase sex from women), were found to be at even higher risk of acquisition of HIV and other sexually transmitted infections (STIs) [4]. Higher risk behaviors like having multiple sexual partners, frequent condom-less anal intercourse (CAI) and alcohol/drug abuse seemed to be the potential reasons [5, 6]. Owing to this increased vulnerability to STIs including HIV, compared to other MSM (non-MSW) who never sold sex to male for money or goods [6], some researchers argued that this subgroup should be considered as a separate sentinel population for HIV surveillance.

Risk of acquisition of HIV and other STIs were found to be driven by a spectrum of biological and behavioral factors. Higher burden of these factors cumulatively resulted in a very high prevalence of HIV and other STIs among MSWs in different parts of the globe [7]. Studies conducted in Cote d’Ivoire, Kenya and India revealed HIV prevalences of 40% or higher among MSWs[7]. Another study conducted in Sydney reported that the prevalence of HIV among MSWs (6.5%) was much less than non-MSW MSM (23.9%), while prevalence of other STIs including syphilis were similar across these two subgroups of MSM [6].

One prior investigation involving MSWs in two cities in China raised similar concern regarding the burden of risky sexual practices in this largely hidden and thus understudied population. Only 53.1% and 70.7% MSWs were found to use condom while engaging in receptive and insertive anal intercourse with clients [8]. Based on studies conducted between 2004–2011, involving non-representative purposive sampling, the prevalence of HIV and syphilis among Chinese MSWs were estimated to be 6.0% and 12.4% respectively [9]. Moreover few studies conducted in China have compared MSWs with non-MSW MSM, regarding socio-demographics, sexual behaviors, STI related symptoms, used of preventive services, knowledge regarding HIV/AIDS along with burden of HIV and other STIs.

The dearth of information regarding comparative evaluation of the burden and correlates of HIV and syphilis between MSWs and non-MSW MSM called for a detailed investigation involving a multisite, representative sample of MSWs. Thus to answer the public health question—whether MSWs are different from non-MSW MSM, in terms of the risk and determinants of acquisition of HIV and syphilis- we analyzed data from a comprehensive, cross-sectional study conducted among MSM from seven metropolitan cities in China during 2008–09.

## Methods

### Recruitment

Respondent-driven sampling (RDS) [10] was used in five urban cities (Nanjing, Chongqing, Jinan, Haerbin, and Guangzhou), while snowball sampling [11] was used in Yangzhou and Suzhou). Males, aged 18 years or more, who were engaged in oral/anal sex with men during the last 12 months and provided written informed consent were eligible for the study. Subjects who had participated in any other similar survey during the previous three months were excluded [12].

### Measures

After the collection of inform consents and biological samples, a structured questionnaire was administered by a trained interviewer to collect information through a face-to-face interview regarding socio-demographics, sexual behaviors (including sexual behaviors with both male and female) and services received regarding prevention/management of HIV and other STIs.

Socio-demographic information included age (continuous and categorized into < 20/ 20-29/30-39/≥40 years), educational level (primary school and below/junior or senior high school/ college and above), marital status (never married/ever married), residence (city where recruited/other city in the same province where recruited/other provinces, while subjects residing in the later two were considered to be migrants), race (Han/others), annual income (≤2000/2001-6000/≥6000 USD), social network size (continuous and categorized into 1, 2–10 and >10) and usual venues for finding male partners (pub, disco or tearoom/spa or bathhouse/park or public restroom/internet and others). Self-identified sexual orientation of the participants (homosexual/heterosexual/bisexual and undecided) was also requested.

Information on sexual behaviors of the participants, condom use during last anal sex (yes/no), consistent condom use (with male and female, yes/no) during last six months, number (continuous) of different male partners in past six months and history of selling anal sex to men during last six months (yes/no) was gathered. Condom-less anal intercourse (CAI) was defined as inconsistent condom use during anal intercourse in previous six months with all male partners, CAI during last anal intercourse was defined as non-use of condoms during last anal intercourse with a male partner and condom-less vaginal intercourse (CVI) was defined as inconsistent condom use during vaginal intercourse in the previous six months with all female partners. Male commercial sex workers were defined as the participants who sold anal sex to men in the past six months, either for money, goods or other benefits (like getting shelter).

Each of the participants was also interviewed to collect information regarding history of experiencing any symptom of STI (burning pain while urinating, genital discharge or ulcer/sores on penis/anus) during the last year, knowledge regarding HIV (correct answer for at least six questions out of eight was defined as correct knowledge) and history of receiving any kind of HIV related service in the last year.

### Serologic measures

Before the interview, five ml of venous blood was collected from each participant for HIV and syphilis testing. HIV antibodies were screened using a rapid test (Acon Biotech Co., Ltd, lot 200803973/WB). Screened positive samples were further confirmed by Western blot (HIVBLOT 2.2, Genelabs Diagnostics, Singapore, lot AE8039). Syphilis antibodies were screened using Rapid Plasma Reagin (RPR; Beijing WanTai Biological Pharmacy Enterprise Co., Ltd., lot N20080404) test and those individuals with a positive RPR titer received the Treponema Pallidum Particle Agglutination assay (TPPA; Livzon Group Reagent Factory, Guangdong, China, lot VN80803). Syphilis positivity was deemed “current” when both TPPA and RPR assays were positive.

### Data Analysis

Data (S1 Dataset) were double-entered using the software EpiData 3.0 [13] by the local centers for diseases prevention and control (CDCs). After the collection of data from all CDCs further logic checks were performed centrally. Identified errors were corrected by communicating with the local CDC and re-checking the original questionnaires. SAS version 9.1 [14] was used for all statistical analyses. Descriptive analyses were conducted to determine the distribution of the demographic factors, behaviors, HIV and syphilis prevalence and other related information for both MSWs and non-MSW MSM. To identify the predictors of commercial sexual behavior among the participants, simple bivariate logistic regression analyses [Odds ratios (OR) and corresponding 95% confidence intervals (CI)] were conducted. To control for potential confounders, multiple logistic regressions were further performed to determine adjusted ORs (aOR) and corresponding 95% CIs. Sampling city (seven cities), annual income, social network size and race were included in these multiple logistic regression models.

### Ethics Statement

Signed informed consent was obtained from each of the participants prior to interview, blood collection and intervention. Each of the participants had the ability to withdraw from this survey at any time after recruitment. The questionnaires and written consent document were separately kept in locked cupboards at the study sites, and unauthorized persons were unable to access them. The study protocol, procedures and content were reviewed and approved by the Ethics Committee of the China CDC in Beijing [15].

## Results

### Descriptive analyses (Table 1)

Altogether 2618 participants were recruited in this comprehensive survey involving MSM from seven urban cities in China between 2008 and 2009. About 59.13% were aged between 20 and 29 years, 74.60% were never married, 50.19% resided in the city from which they were recruited, 97.67% had Han ethnicity, 49.24% attended junior or senior high school and 56.19% self-identified themselves as homosexuals.

Participants identified their male sexual partners most commonly through internet (55.84%) followed by pubs/clubs (14.40%). Social network size was only one for 12.18% subjects, while 48.93% had network sizes larger than 10. During the last year, 69.10% of participants used some kind of STI related services. HIV related knowledge was correct for 79.37% and 19.81% experienced some STI related symptoms during last year.

A total of 261 (9.97%) participants reported that they sold sex to other males in past six months, 33.94% did not use a condom during their last anal sex with a male partner (CAI in last anal intercourse), 55.04% reported that they had CAI during the last six months and 18.68% engaged in CVI in the last six months.

In this study, 195 HIV positive MSM were identified through lab testing, with an overall prevalence of 7.45% [6.13% (16/261) for MSWs and 7.59% (179/2357) for non-MSW MSM, *P* = 0.39]. Among participating subjects, 375 were currently sero-positive for syphilis, with a prevalence of 14.32% [10.73% (28/261) for MSWs and 14.72% (347/2357) for non-MSW MSM, *P* = 0.08].

Detailed information regarding socio-demographics, behaviors and burden of HIV and syphilis for both MSWs and non-MSW MSM are also presented in Table 1.

**Table 1:** Distribution of socio-demographics, sexual behavior and HIV/syphilis prevalence among men who have sex with men in China, 2008–2009 (N = 2618).

Variables		MSWs (n = 261)				Other MSM (n = 2357)				Overall (n = 2618)	
		Frequency	Percent	95% CL lower	95% CL upper	Frequency	Percent	95% CL lower	95% CL upper	Frequency	Percent
**Age**	*Less than 20*	43	16.48	11.95	21.01	110	4.67	3.81	5.52	153	5.84
	*20–29*	179	68.58	62.91	74.25	1369	58.08	56.09	60.08	1548	59.13
	*30–39*	28	10.73	6.95	14.51	500	21.21	19.56	22.87	528	20.17
	*40 and above*	11	4.21	1.76	6.67	378	16.04	14.55	17.52	389	14.86
**Marital status**	*Never married*	236	90.42	86.83	94.02	1717	72.85	71.05	74.64	1953	74.6
	*Ever married*	25	9.58	5.98	13.17	640	27.15	25.36	28.95	665	25.4
**Residence**	*Sampling city*	70	26.82	21.41	32.23	1244	52.78	50.76	54.8	1314	50.19
	*Same province as sampling city*	85	32.57	26.84	38.29	543	23.04	21.34	24.74	628	23.99
	*Other provinces*	106	40.61	34.62	46.61	570	24.18	22.45	25.91	676	25.82
**Race**	*Han*	258	98.85	97.55	100	2299	97.54	96.91	98.17	2557	97.67
	*Others*	3	1.15	0	2.45	58	2.46	1.83	3.09	61	2.33
**Education**	*Primary school or below*	15	5.75	2.9	8.59	47	1.99	1.43	2.56	62	2.37
	*Junior or senior high school*	206	78.93	73.95	83.91	1083	45.95	43.93	47.96	1289	49.24
	*College or above*	40	15.33	10.93	19.72	1227	52.06	50.04	54.08	1267	48.4
**Annual income**	*2000 and below*	81	31.03	25.38	36.68	733	31.1	29.23	32.97	814	31.09
	*2001–6000*	133	50.96	44.85	57.06	1200	50.91	48.89	52.93	1333	50.92
	*Above 6000*	47	18.01	13.32	22.7	424	17.99	16.44	19.54	471	17.99
**City which recruited**	*Nanjing*	24	9.2	5.67	12.72	355	15.06	13.62	16.51	379	14.48
	*Suzhou*	65	24.9	19.62	30.19	190	8.06	6.96	9.16	255	9.74
	*Yangzhou*	35	13.41	9.25	17.57	213	9.04	7.88	10.2	248	9.47
	*Chongqing*	49	18.77	14.01	23.54	464	19.69	18.08	21.29	513	19.6
	*Guangzhou*	7	2.68	0.71	4.65	329	13.96	12.56	15.36	336	12.83
	*Haerbin*	30	11.49	7.6	15.39	357	15.15	13.7	16.59	387	14.78
	*Ji'nan*	51	19.54	14.7	24.38	449	19.05	17.46	20.64	500	19.1
**Knowledge**	*No*	66	25.29	19.98	30.6	474	20.11	18.49	21.73	540	20.63
	*Yes*	195	74.71	69.4	80.02	1883	79.89	78.27	81.51	2078	79.37
**Received HIV related service**	*No*	77	29.5	23.93	35.07	732	31.06	29.19	32.93	809	30.9
	*Yes*	184	70.5	64.93	76.07	1625	68.94	67.07	70.81	1809	69.1
**Venue**	*Pub*, *Disco*, *Tearoom*, *or Club*	95	36.4	30.52	42.27	282	11.96	10.65	13.28	377	14.4
	*Spa or bathhouse*	30	11.49	7.6	15.39	268	11.37	10.09	12.65	298	11.38
	*Park or Public Restroom*	39	14.94	10.59	19.3	246	10.44	9.2	11.67	285	10.89
	*Internet*	84	32.18	26.48	37.89	1378	58.46	56.47	60.46	1462	55.84
	*Others*	13	4.98	2.32	7.64	183	7.76	6.68	8.85	196	7.49
**Sexual orientation**	*Homosexual*	125	47.89	41.79	53.99	1346	57.11	55.11	59.11	1471	56.19
	* Heterosexual*	19	7.28	4.11	10.45	14	0.59	0.28	0.9	33	1.26
	* Bisexual*	100	38.31	32.38	44.25	876	37.17	35.21	39.12	976	37.28
	* Undecided*	17	6.51	3.5	9.53	121	5.13	4.24	6.03	138	5.27
**Experienced STI related symptoms in last year**	*Yes*	44	30.34	22.77	37.92	325	18.92	17.06	20.77	369	19.81
	*No*	101	69.65	62.08	77.23	1393	81.08	79.23	82.94	1494	80.19
**Social network size**	*One*	47	18.01	13.32	22.7	272	11.54	10.25	12.83	319	12.18
	*2 to 10*	97	37.16	31.26	43.07	921	39.08	37.1	41.05	1018	38.88
	*above 10*	117	44.83	38.75	50.9	1164	49.38	47.37	51.4	1281	48.93
**CAI in last six months**	*Yes*	142	54.41	48.32	60.49	1299	55.11	53.1	57.12	1441	55.04
	*No*	119	45.59	39.51	51.68	1058	44.89	42.88	46.9	1177	44.96
**CAI in last anal intercourse**	*No*	198	76.45	71.24	81.65	1462	64.86	62.89	66.83	1660	66.06
	*Yes*	61	23.55	18.35	28.75	792	35.14	33.17	37.11	853	33.94
**CVI **	*Yes*	74	28.35	22.85	33.86	415	17.61	16.07	19.14	489	18.68
	*No*	187	71.65	66.14	77.15	1942	82.39	80.85	83.93	2129	81.32
**HIV**	*Positive*	16	6.13	3.2	9.06	179	7.59	6.52	8.66	195	7.45
	*Negative*	245	93.87	90.94	96.8	2178	92.41	91.34	93.48	2423	92.55
**Syphilis**	*Positive*	28	10.73	6.95	14.51	347	14.72	13.29	16.15	375	14.32
	*Negative*	233	89.27	85.49	93.05	2010	85.28	83.85	86.71	2243	85.68

### Correlates of commercial sexual behavior (Comparison between MSWs and non-MSW MSM) (Table 2)

In the bivariate analyses (unadjusted models), likelihood of engaging in commercial sex was found to decrease significantly with increasing age (OR = 0.91, 95% CI: 0.89–0.91). MSWs were less likely (OR = 0.74, 95% CI: 0.55–1.00) to have correct HIV related knowledge, to engage in CAI during last anal intercourse (OR = 0.57, 95% CI: 0.42–0.77) and CAI in last six months (OR = 0.97, 95% CI: 0.75–1.26) although the results for the CAI in last six months were not statistically significant.

**Table 2:** Factors correlated with male sex work (MSWs) in China, 2008–2009 (N = 2618).

Variables		Unadjusted Model			Adjusted model*		
		OR	95% CI	*P*-value	AOR	95% CI	*P*-value
**Age**	** **	0.91	0.89,0.93	<0.001	0.91	0.88,0.93	<0.001
**Marital status**	*Ever marred*	*Ref*	* *	* *	*Ref*		
	*Never married*	3.52	2.31,5.37	<0.001	4.38	2.83,6.8	<0.001
**Residence**	*Sampling city*	*Ref*	* *	* *	*Ref*		
	*Some province of sampling city*	2.78	2.00,3.88	<0.001	1.15	0.88,1.49	0.30
	*Other provinces*	3.31	2.40,4.54	<0.001	1.13	0.86,1.48	0.37
**Education**	*Primary school or below*	9.79	5.06,18.96	<0.001	13.41	6.60,27.24	<0.001
	*Junior or senior high school*	5.84	4.12,8.27	<0.001	6.96	4.77,10.14	<0.001
	*College or above*	*Ref*	* *	* *	*Ref*		
**Venue**	*Pub*, *Disco*, *Tearoom*, *or Club*	5.53	4.01,7.61	<0.001	5.01	3.55,7.08	<0.001
	*Spa or bathhouse*	1.84	1.19,2.84	0.01	1.76	1.10,2.80	0.02
	*Park or Public Restroom*	2.60	1.74,3.89	<0.001	3.12	1.98,4.94	<0.001
	*Internet*	*Ref*	* *	* *	*Ref*		
	*Others*	1.17	0.64,2.13	0.62	1.15	0.62,2.12	0.66
**Sexual orientation**	*Homosexual*	*Ref*	* *	* *	*Ref*		
	*Heterosexual*	14.61	7.15,29.85	<0.001	13.04	6.08,27.95	<0.001
	*Bisexual*	1.23	0.93,1.62	0.14	1.07	0.80,1.43	0.67
	*Undecided*	1.51	0.88,2.60	0.13	1.69	0.96,2.97	0.07
**Knowledge**	*No*	*Ref*	* *	* *	*Ref*		
	*Yes*	0.74	0.55,1.00	0.05	0.70	0.51,0.96	0.03
**Received HIV related service**	*No*	*Ref*	* *	* *	*Ref*		
	*Yes*	1.08	0.81,1.42	0.61	1.13	0.84,1.52	0.43
**STIs related symptoms in the last year**	*No*	*Ref*	* *	* *	*Ref*		
	*Yes*	1.87	1.28,2.71	<0.001	2.16	1.47,3.19	<0.001
**CAI in last six months**	*No*	*Ref*	* *	* *	*Ref*		
	*Yes*	0.97	0.75,1.26	0.83	0.91	0.70,1.19	0.50
**CAI during last anal intercourse**	*No*	*Ref*			*Ref*		
	*Yes*	0.57	0.42, 0.77	<0.001	0.62	0.46, 0.85	0.003
**CVI**	*No*	*Ref*					
	*Yes*	1.85	1.39,2.47	<0.001	1.55	1.13, 2.11	0.006
**HIV**	*Negative*	*Ref*	* *	* *	*Ref*		
	*Positive*	0.80	0.47,1.35	0.39	0.72	0.42,1.24	0.23
**Syphilis**	*Negative*	*Ref*	* *	* *	*Ref*		
	*Positive*	0.70	0.46,1.05	0.08	0.66	0.43,1.01	0.05

Note: * Model adjusted for city (seven cities), monthly income (≤2000/2001-6000/≥6000 USD),), social network size (1, 2–10, above 10) and Race (han, others)

Compared to non-MSW MSM, MSWs were more likely to be unmarried (OR = 3.52, 95% CI: 2.31–5.37), migrants (residents of other cities in the same province or other provinces), engaged in commercial sex with males [having unadjusted ORs of 2.78 (95% CI: 2.00–3.88) and 3.31 (95% CI: 2.40–4.54), respectively]. They also had relatively less education, as evidenced from the unadjusted ORs of 5.84 (95% CI: 4.12–8.27) and 9.79 (95% CI: 5.06–18.96), respectively for junior/senior high school and elementary school or less education compared to those who were at least educated up to college level.

Odds of finding male partners on the internet, in pubs/disco, spa/bathhouse and park/public restrooms were also higher for MSWs (relative to the non-MSW MSM), with unadjusted ORs of 5.53 (95% CI: 4.01–7.61), 1.84 (95% CI: 1.19–2.84) and 2.60 (95% CI: 1.74–3.89), respectively. MSWs were also more likely to be self-identifying heterosexuals, with unadjusted OR of 14.61 (95% CI 7.15–29.85).

With reference to their non-MSW counterparts, MSWs had a lower HIV prevalence, although the result was not significant. MSWs were also less likely to be syphilis positive (unadjusted OR = 0.70, 95% CI: 0.46–1.05) but more likely to have symptoms suggestive of STIs during the last year, unadjusted OR of 1.87 (95% CI: 1.28–2.71).

After adjustment for recruitment site, monthly income, social network size and race, using multiple logistic regression models, the results did not change much, except for residence, which was no longer associated with commercial sexual behaviors in our study. In the adjusted model, compared to non-MSWs, MSWs were more likely to be younger (aOR = 0.91, 95%CI = 0.88–0.93), never married (aOR = 4.38, 95% CI = 2.38–6.80, reference = ever married) and less educated (for those educated up to primary school, aOR = 13.41, 95% CI = 6.60–27.24 and for those educated up to high school, aOR = 6.96, 95% CI = 4.77–10.14, reference = educated up to college or above). They also had higher odds of being heterosexual (aOR = 13.04, 95% CI = 6.08–27.95, reference = homosexual), less knowledgeable regarding HIV (aOR = 0.70, 95% CI = 0.51–0.96, reference = having incorrect knowledge), seeking male sexual partners usually in pub/disco/tearoom/club (aOR = 5.01, 95% CI = 3.55–7.08, reference = internet) and park/public restroom (aOR = 3.12, 95% CI = 1.98–4.94), experiencing symptoms of STI (aOR = 2.16, 95% CI = 1.47–3.19, reference = no). With reference to the non-MSW MSM, MSWs engaged more commonly in unprotected vaginal intercourse (aOR = 2.16, 95% CI = 1.47–3.19, reference = no) but less often in unprotected anal intercourse (aOR = 0.62, 95% CI = 0.46–0.85, reference = no).

## Discussion

The burden of HIV among MSWs across the globe warranted a comparative evaluation of the burden and correlates of HIV and syphilis between MSWs and their non-MSW counterparts in China where such comparisons were conducted rarely [7]. The findings from this multicenter study confirmed that the prevalence of HIV and syphilis were high among MSWs in urban China. The distribution of the potential socio-demographic and behavioral risk factors of HIV and syphilis varied considerably across these two subgroups of the understudied MSM in China.

In this survey involving a multicentre population of urban MSM of China the prevalence of HIV and syphilis were found to be slightly lower among MSWs compared to their non-MSW counterparts although the difference was not statistically significant for HIV. Our study further confirmed the results of one meta-analysis that focused on Chinese MSM[16]. Similar observations were also reported by one study conducted at Sydney, where significantly lower prevalence of HIV was revealed among MSWs than the non-MSW MSM [6]. A few factors could lead to this seemingly contradictory result. First, although our study indicated that proportion of MSWs having CAI in the last six months were similar to that among non-MSW MSM, MSWs had significantly lower proportions for CAI during their last anal intercourse. The lower burden of this important risk behavior might well have reduced the risk of acquisition of HIV and syphilis infection among MSWs, compared to non-MSW MSM despite the fact that MSWs had relatively much higher number of sexual partners (22.49±6.59 VS 4.00 ±3.35 in the last six months). Second: very few MSWs in China might have identified themselves as sex workers. They probably belonged to a group of younger men with lower socio-economic status (SES) who offered sex to other male in exchange for money, gifts or other economic support, and were often not even gay or bisexual. They might also have worked as MSWs for only a short period of time to earn some quick money [7]. Data collected from our study also supported these explanations, as the MSWs in our study were more likely to be younger, less educated, migrants and heterosexual. Third: as a cross-sectional study, our study might have suffered from some selection bias, particularly if the response rate among different sub-groups remained different and was influenced by their commercial sexual behavior The participating MSWs might be belonging to a more aware proportion of the population they were representing, who could have relatively better health-seeking and less risk behavior compared to those who did not participate.

The non-consistent results between CAI in the last six months and CAI during last anal intercourse might have indicated that CAI during last anal intercourse could be a more sensitive way to measure the behavior regarding getting engaged in CAI among MSM.

Although MSWs had a slightly lower syphilis prevalence compared to non-MSW MSM, relatively higher proportion of them experienced STI related symptoms in the last year. This finding emphasized the potentially higher risk of recurrent STIs among MSWs.

MSWs were typically younger, less likely to be married, received less education and were more often migrants. These characteristics probably indicated that the SES of MSWs in China was poor in general. These findings were likely to explain the relative lack of knowledge regarding HIV among MSWs. All of these factors were found in previous studies to enhance the spread of HIV, syphilis and other STIs in different high risk population including MSM and MSWs seemed to be no exception. They were probably more vulnerable owing to poorer self-esteem and resultant self-neglect [17]. The strengths of our study included the multicenter study design, a large sample size and non-perspective or opportunistic sampling methods (RDS and snowball) which might have resulted in a more a representative population of urban MSM in China, although no sampling method is likely to recruit a completely representative people of this hidden population. Our study also had several potential limitations. The data was collected between 2008 and 2009, and may not represent population characteristics of 2015. Like all cross-sectional studies, our study might have suffered from some selection bias [18] and also lacked temporality. Thus, all the correlations identified in our study should be interpreted with caution and causal inferences are not recommended. Most of the information collected in our study was based on the self-report of the participants through face-to-face interview, which might have introduced some social desirability issues. Moreover, we did not collect detailed information on type (insertive or receptive) of anal intercourse and frequency of condom, which limited our ability to further explore the differences between MSWs and non-MSWs MSM on these variables.

Despite these limitations, this comprehensive multicenter study suggests that HIV and syphilis prevalence among MSWs and non-MSWs MSM is high in urban China. MSWs differed from non-MSWs in terms of SES, sexual orientation, usual venues for finding sexual partners, high risk behaviors, syphilis prevalence and STI related symptoms. Further studies should be conducted to explore these differences in details. Although male sex workers and non-commercial homosexuals have similar rates of HIV and syphilis, MSWs have different characteristics which should be considered in designing intervention programs targeting them.

## Supporting Information

S1 Dataset (XLS)
